# A Ranking Recommendation Algorithm Based on Dynamic User Preference

**DOI:** 10.3390/s22228683

**Published:** 2022-11-10

**Authors:** Chunting Wei, Jiwei Qin, Qiulin Ren

**Affiliations:** 1 School of Information Science and Engineering, Xinjiang University, Urumqi 830046, China; 2 Key Laboratory of Signal Detection and Processing, Xinjiang Uygur Autonomous Region, Xinjiang University, Urumqi 830046, China

**Keywords:** recommender systems, collaborative filtering, attention, deep learning, dynamic preference modeling

## Abstract

In recent years, hybrid recommendation techniques based on feature fusion have gained extensive attention in the field of list ranking. Most of them fuse linear and nonlinear models to simultaneously learn the linear and nonlinear features of entities and jointly fit user-item interactions. These methods are based on implicit feedback, which can reduce the difficulty of data collection and the time of data preprocessing, but will lead to the lack of entity interaction depth information due to the lack of user satisfaction. This is equivalent to artificially reducing the entity interaction features, limiting the overall performance of the model. To address this problem, we propose a two-stage recommendation model named A-DNR, short for Attention-based Deep Neural Ranking. In the first stage, user short-term preferences are modeled through an attention mechanism network. Then the user short-term preferences and user long-term preferences are fused into dynamic user preferences. In the second stage, the high-order and low-order feature interactions are modeled by a matrix factorization (MF) model and a multi-layer perceptron (MLP) model, respectively. Then, the features are fused through a fully connected layer, and the vectors are mapped to scores. Finally, a ranking list is output through the scores. Experiments on three real-world datasets (Movielens100K, Movielens1M and Yahoo Movies) show that our proposed model achieves significant improvements compared to existing methods.

## 1. Introduction

In the era of information overload, users are faced with massive information choices every day [1]. As a means to solve the information overload on the Internet, recommender systems have received extensive attention and developed rapidly [1]. The recommendation system regards users and items as entities, then abstracts the process of information selection into entity interactions, and finally fits these interactions through various models to predicte users’ needs. Moreover, recommender systems (RSs) have been successfully used commercially by Netflix, YouTube [2] or Amazon. In recent years, with the increasing requirements of users to obtain information preferences, list ranking recommendation has become one of the hottest directions of recommendation systems [3].

The MLP model has the characteristics of flexibility and high capacity in learning high-order feature interactions, but its ability to capture low-order interactions is very limited, while the basic function of the MF model is to capture the linear features of users and items and fit the low-order interactions of entities. Therefore, many recommendation models are keen to combine MLP and MF to fuse linear features and nonlinear features to jointly fit entity interactions. Most of these feature fusion models are based on implicit feedback, that is, the score of entity interaction is set to 1, and the score of no observed entity interaction is set to 0, which can reduce the complexity of data processing and reduce training time. But they ignore a key issue: the contribution of different feature interactions to user preferences may not be the same, that is, user satisfaction is not observed. In real-world applications, different predictor vectors usually have different predictive powers, therefore, less useful feature interactions should be given lower weights because they contribute less to the prediction. In addition, users’ preferences change over time, and recently interacted items can more reflect the user’s future preferences, but historical interactions too long in the past cannot accurately represent the user’s current interests. For example, a user used to watch comedy movies in the past, but now becomes more interested in action movies after a period of time. In this way, a list containing more action movies would not be recommended according to the user’s historical interaction.

To address the above problems, we propose a feature fusion ranking model based on dynamic user preferences, abbreviated as A-DNR. It is a two-stage recommendation model, namely dynamic user preference modeling based on attention mechanism and list ranking recommendation based on feature fusion. In the first stage, users’ long-term preferences are modeled through user history interactions, and then a time-aware attention mechanism network is built through recent interaction items and interaction times to model user short-term preferences. Finally, the generated short-term preferences and long-term preferences are combined into dynamic user preferences and input into the feature interaction layer. In the second stage, low-order and high-order feature interactions are modeled by the MF model and MLP model, respectively. Then feature fusion is performed through a fully connected layer and vectors are mapped to scores. Finally, a ranking list is output through scores. A-DNR fuses linear and nonlinear models and combines their advantages to make more full use of linear and nonlinear features generated by user-item interactions. More importantly, the A-DNR model establishes the user long-term and short-term preferences through the time-aware attention mechanism network, integrates the input layer, and assigns greater weight to the high-value interaction vectors, which can fit the user-item interactions more accurately. In the experimental part, we will conduct extensive experiments to further demonstrate the feasibility and effectiveness of introducing dynamic user preferences.

In summary, our main contributions are as follows:

- We proposed a new neural network architecture based on attention mechanism to fit the linear and nonlinear interaction process of users and items.
- We adopt a new time-aware attention mechanism, which fully considers the historical interaction items, recent interaction items and their interaction time to dynamically model user preferences.
- We explore the effects of parameters such as list length and attention network layer depth on the A-DNR model through extensive experiments.
- We conduct extensive experiments on three real-world datasets to demonstrate the effectiveness of the A-DNR approach and discuss future research directions.

## 2. Related Work

In this section, we briefly introduce some background material and related work. First, we introduce some ranking-oriented traditional methods and deep learning models and analyze their respective advantages and disadvantages. Then, the attention mechanism, time-aware recommender systems, and some classical algorithms based on the attention mechanism are briefly introduced, which inspires our proposed method.

### 2.1. Traditional Ranking Methods

MF is one of the most efficient methods for handling various recommendations [4]. The core idea of MF is to learn a low-dimensional latent feature to represent users and items. Many MF methods focus on prediction accuracy, but low prediction error does not guarantee high-quality recommendations [5,6]. Rendle et al. [7] proposed a Bayesian ranking framework that utilizes users’ implicit feedback (such as clicks, favorites, shopping carts, etc.) to rank items through the maximum posterior probability obtained by Bayesian analysis. Park et al. [8] utilized some auxiliary information, such as user statistics and item content features, to solve the cold start problem. Shi et al. [9] proposed a method ListRank-MF that combines a list-based learning ranking algorithm with matrix factorization (MF), which optimizes the model for recommendation by minimizing a loss function.

Although these methods perform well, they suffer from a key problem: they all employ matrix factorization and inner products and cannot fully capture complex structures and deeper information from user-item interactions. Traditional methods represented by MF only consider linear interactions between users and items, without exploring nonlinear and more complex interactions between them.

### 2.2. Ranking Methods Based on Deep Learning

In recent years, deep learning models have achieved great success in machine translation, image recognition, etc. due to their powerful ability to learn complex nonlinear representations through hidden layers [10]. Many studies have shown that with enough hidden layers and neural units, DNNs can approximate any continuous function with arbitrary precision [11,12]. Recommendations using DNNs have also become more popular in recent years. For example, Chen et al. [13] proposed the DeepRank ranking model, which uses an MLP composed of DNNs to model higher-order interactions of entities and improve the quality of personalized ranking. Suvash et al. [14] built an AutoEncoder (AE) model through DNN to predict the missing rating values in the user-item matrix. Subsequently, Vincent et al. [15] proposed the Denoise Autoencoder (DAE) model, which uses the corrupted input data to reconstruct the original data to enhance the robustness of the training features. Wu et al. [16] proposed an improved method, Collaborative Denoising Auto-Encoders (CDAE), by integrating user-specific biases into DAE to capture the latent features of items.

Deep learning models can capture complex and in-depth information about user-item interactions and enrich the deep representations of users and items. However, the single use of deep learning models to fit user-item interactions limits the learning of linear interactions. This is a considerable loss to the expressiveness of the entire model.

### 2.3. Hybrid Recommendation Methods Based on Feature Fusion

Recently, many hybrid recommendation models with the ability to capture linear and nonlinear features have emerged. For example, Cheng et al. [17] designed the Wide&Deep framework that integrates the wide model and the deep model for joint training, which comprehensively utilizes the memory ability of the linear model and the generalization ability of the nonlinear model to achieve the accuracy and expansion of the model to the recommendation system. gender balance. Guo et al. [10] proposed a Factorization-Machine based neural network (DeepFM) model, which improved the ability to extract information on the wide side of the model by replacing the linear model in the Wide&Deep framework with a Factorization Machine (FM). He et al. [18] proposed the Neural Factorization Machine (NFM) model, which processed the second-order cross-information by introducing the Bi-linear interaction structure into the Wide&Deep framework, which reduced the difficulty of DNN learning higher-order cross-feature information and reduced the Model training time. Deng et al. [19] proposed the Deep Collaborative Filtering (DeepCF) model. By combining the methods based on representation learning and learning based on matching functions, the model endows the model with the ability to learn linear and nonlinear features of entities. He et al. [20] used a neural network to convert the MF model into a DMF model and then fused it with the MLP, and proposed the NeuMF model. Kim et al. [21] improved the NeuMF model by replacing the original MLP with a convolutional neural network (CNN) and proposed a convolutional matrix factorization (ConvMF) model. 

These models capture the linear and nonlinear features of entities through linear and nonlinear models respectively, and fit the linear and nonlinear interactions of entities to improve recommendation quality. However, they are usually trained with one-hot encoded binarized sparse features, setting a score of 1 for interactions in the user-item interaction matrix and 0 otherwise. This will lead to the lack of in-depth interactive information, which makes the model unable to accurately learn user preferences and ultimately limit the overall performance of the model.

### 2.4. Time-Aware Recommender Systems

In recent years, more and more researchers time-aware recommender systems. Koren et al. [22] Proposed the TimeSVD++ model. It is a well-known time-aware recommender model that introduces time-variant bias for each user and item at each time step to improve SVD++, which is the champion algorithm of the Netflex Prize. Li et al. [23] Proposed the neural attentive recommendation machine (NARM) model. It uses the RNN and the attention network to model users’ general interests and main purpose, respectively. Zhou et al. [24] Proposed the ATRank model. It introduces the multi-head self-attention mechanism in Transformer for rating predictions, which can speed up the training speed and improve the prediction accuracy. Yu et al. [25] Proposed the MARank model. It is a multi-level attentive ranking model, which can unify individual- and union-level item interactions into preference modeling. Individual-level interaction highlights the transition order between item, and union-level interaction represents the relation between a single item and a group of items. Wang et al. [26] proposed the time-aware attention-based deep collaborative filtering (TADCF) model, which predicts the missing interactions through time-aware attention mechanisms and different types of DL models.

The ability of these models for time simulation is not prominent enough, and they can not well integrate user long-term and short-term preferences.

### 2.5. Recommendation Method Based on Attention Mechanism

In recent years, more and more researchers have begun to use attention mechanisms to model user preferences, and have achieved advanced performance [27]. Xiao et al. [28] first proposed an attentional network-based multimedia CF recommendation model, Attentional Factorization Machines (AFM). It seamlessly integrates the attention mechanism into the classic CF model, using two attention models to capture the importance of different interaction items and different components of items, and is one of the most classic and popular algorithms at present. He et al. [29] proposed an item-based CF model, Neural Attentive Item Similarity model (NAIS), which models the importance of user preferences using different historical items in an attention network. In the NAIS model, the attention network provides NAIS with a stronger representation than traditional CF methods at the cost of only a few extra parameters. Ze et al. [30] proposed the Deep Match to Rank (DMR) model, which integrates matching tasks and ranking tasks into a unified model for click-through rate (CTR) prediction. Zhang et al. [31] proposed the Dynamic network embedding via structural attention (DNESA) model, which combines the attention mechanism with the network embedding to focus on task-relevant parts and avoid noisy parts in the network.

These recommendation methods use the attention mechanism to model long-term user preferences based on the CF algorithm and achieve more advanced performance. To a certain extent, these methods have also greatly inspired our proposed model. However, user preferences change over time, and past interactions can’t represent current user preferences. These models ignore the time factor when considering user preferences, so the obtained user preferences are static, which will limit the performance of the model.

## 3. Our Approach

We first show the overall architecture and operating principle of the A-DNR framework, and focus on the analysis of attention layer and feature interaction layer. Then, we demonstrate how to use the A-DNR model to rank and how to train the model.

### 3.1. Overall Architecture

**Figure 1:**
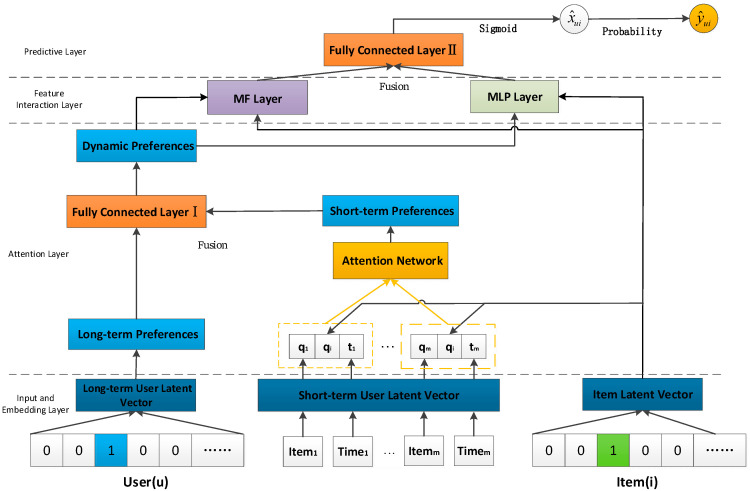
The overall architecture of the A-DNR framework.

Figure 1 shows the overall architecture of the A-DNR model. A-DNR is a two-stage recommendation model with historical interaction matrix R, recent interaction matrix $R^{*}$ and the interaction time Y of $R^{*}$ as inputs. In the first stage, an implicit interaction matrix R is used to encode user and item embeddings. Since the interaction matrix R represents the user’s historical interactions with items, according to models such as DNESA, user embeddings can be viewed as user long-term preferences. Meanwhile, user short-term preferences can be captured using the attention network through historical interaction items and their interaction time. In this way, dynamic user preferences can be modeled by combining short-term and long-term user preferences. Finally, dynamic user preferences are input into the MF and MLP layers as user embedding vectors along with item embedding vectors. In the second stage, the MF layer and the MLP layer process the output of the attention network respectively, and then the two are weighted through the fully connected layer to obtain the matching score. Finally, each item in the list can be sorted by mapping the scores to probabilities. Next, we will focus on the attention layer and the feature interaction layer.

### 3.2. Attention Layer

Generally, user preferences can be roughly divided into long-term and short-term [32]. Long-term preferences often refer to the interests of users. For example, if a user is a fan of Tom Hardy, she will be interested in Tom Hardy’s movies and promotional videos for a long time in the future. This is also the direction that most attention-based models focus on, that is, modeling user preferences through all items in the user interaction history, and mapping the characteristics of historical interaction items to long-term preferences. But user preferences are not static, and items interacted with in the past may not accurately reflect current user interests. For example, if a user who previously liked Tom Hardy recently became a fan of Jason Statham, a sorted list obtained through long-term preferences may not rank Jason’s movies first. Therefore, the focus of our work is to capture users’ short-term preferences and fuse them with long-term preferences to obtain dynamic user preferences for list ranking.

**Figure 2:**
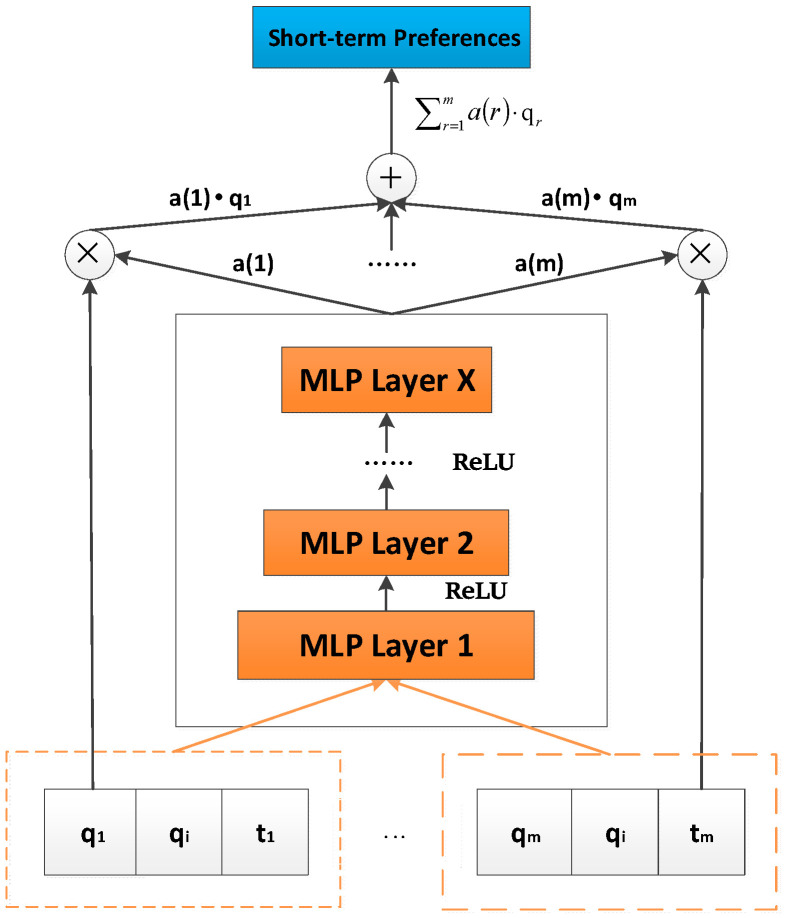
The architecture of the attention network.

Figure 2 shows the capture process of user short-term preferences. We integrate the embedding vector $q_{i}$ of the predicted item i, the embedding vector $q_{r}$ of the most recent interaction item r and its interaction time vector $t_{r}$ into a vector group $\left(q_{r},q_{i},t_{r}\right)$ as the input of the attention network. The weights for each interaction item are learned by MLP as follows:

(1) $$O_{0}=\left(q_{r},q_{i},t_{r}\right)$$

(2) $$O_{1}=k_{1}\left(O_{0}\right)=\alpha_{1}\left(h_{1}^{T}O_{0}+c_{1}\right)$$

(3) $$O_{2}=k_{2}\left(O_{1}\right)=\alpha_{2}\left(h_{2}^{T}O_{1}+c_{2}\right)$$

……

(4) $$O_{L}=k_{L}\left(O_{L-1}\right)=\alpha_{L}\left(h_{L}^{T}O_{L-1}+c_{L}\right)$$

where $O_{0}$ represents the input of the attention network; $\alpha_{L}$, $h_{L}$, $c_{L}$, $O_{L}$ represent activation function, weight matrix, bias and the output of the Lth layer of the attention mechanism network, respectively. According to the literature [17,18,19,20], the ReLU function is more suitable for sparse data, so we choose the ReLU function as the activation function here. For consistency, we use the ReLU function in each hidden layer of the entire model, which will not be repeated below. Finally, the output of the attention mechanism network is as follows:

(5) $$a\left(r\right)=softmax\left(O_{out}\right)$$

where a(r) represents the attention weight of the most recent interaction item r; $O_{out}$ and $h_{out}$ represent the output and weight matrix of the output layer of the attention mechanism network, respectively; $N^{m}\left(u\right)$ represents the m recent interaction items of user u.

Through the attention weights of recent historical interaction items, we can obtain the user short-term preferences as follows:

(6) $$A_{u}^{S}=\sum_{r=1}^{m}a\left(r\right)q_{r}$$

where $A_{u}^{S}$ represents the short-term preferences of user u. Next, the user long-term preference is modeled as the arithmetic average of the user’s embedded vector in the potential space, as follows:

(7) $$A_{u}^{L}=\frac{\sum_{i\epsilon l\left(u\right)}q_{i}}{\left|l\left(u\right)\right|}$$

where l(u) represents all historical interaction items of the user u. Finally, we need to integrate the long-term and short-term preferences of user u. A simple method is to linearly combine the long-term and short-term preferences, and determine the combination weight coefficient through a large number of experiments. The idea is as follows:

(8) $$A_{u}=\mu A_{u}^{S}+\left(1-\mu \right)A_{u}^{L}$$

where $A_{u}$ represents the dynamic preference of user u, and μ∈[0, 1] represents the combination coefficient.

However, this method ignores a problem: not all users have the same long-term and short-term preference weight. For example, user A is a person with changeable styles, and his interests and hobbies change greatly. At this time, we should pay more attention to the short-term preferences of A. For the relatively conservative user B, his interest will not change. At this time, the long-term preference of B should be given greater weight. To sum up, the linear combination method can not accurately capture the dynamic user preferences. To solve this problem, we use a full connection layer to connect long-term and short-term preferences in parallel, and dynamically learn the fusion weight through neural networks. The idea is as follows:

(9) $$A_{u}=\partial \left(H_{1}^{T}\left[\begin{array}{c} A_{u}^{L}\\ A_{u}^{S} \end{array}\right]\right)$$

where $H_{1}$ and α represent the weight matrix and activation function of full connection layer I, respectively.

### 3.3. Feature Interaction Layer

The user’s dynamic preferences are captured through the attention layer. Then, the dynamic user preferences are regarded as user embedding and input into the feature interaction layer together with the item embedding for feature fusion. The feature interaction layer is the fusion of linear method MF and nonlinear method MLP, which makes full use of the linear and nonlinear features of entities to improve the sorting quality. The output of the MF model is as follows:

(10) $$x^{MF}=\left(G_{u}A_{u}^{F}\right)⊙\left(G_{i}q_{i}^{F}\right)$$

where $x^{MF}$ represents the output of the MF model; $G_{u}$ and $G_{i}$ are mapping matrices, which are used to obtain user embedding vectors and item embedding vectors with the same dimension; $A_{u}^{F}$ and $q_{i}^{F}$ represent the dynamic user preference vector and item embedding vector input into the MF model, respectively; ⊙ represents the element product. To fuse the outputs of MLP and MF accurately, we use element product instead of inner product to keep them in the same dimension.

At the same time, the dynamic user preference and item embedding are input into the MLP model to obtain the nonlinear representation of the entity. Its operation process is as follows:

(11) $$x_{1}=k_{1}\left(p_{u},q_{i}\right)$$

(12) $$x_{2}=k_{2}\left(x_{1}\right)=a_{2}(W_{2}^{T}x_{1}+b_{2})$$

……

(13) $$x_{L}=k_{L}\left(x_{L-1}\right)=a_{L}(W_{L}^{T}x_{L-1}+b_{L})$$

where $W_{L}$, $b_{L}$ and $a_{L}$ represent the weight matrix, bias vector and activation function of the L-th layer perceptron, respectively. The output of the MLP hidden layer is defined as:

(14) $$x^{MLP}=a_{L}(W_{L}^{T}\left(\ldots \left(a_{2}W_{2}^{T}\left[\begin{array}{c} A_{u}^{M}\\ q_{i}^{M} \end{array}\right]+b_{2}\right)\ldots \right)b_{L}$$

where $A_{u}^{M}$ and $q_{i}^{M}$ represent the dynamic user preference and item embedding input into the MLP layer, respectively.

Inspired by methods such as NeuMF and DeepCF, we use a parallel approach to fuse MF and MLP models and connect the outputs of the two models using a full connection layer that automatically assigns different weights to features contained in the joint representation. Since these two models have different advantages and learn prediction vectors from different perspectives, the fusion of the MF layer and MLP layer will produce more accurate prediction values and more competitive model performance. The predicted score after fusion are as follows:

(15) $${\hat{x}}_{ui}=\sigma \left(H_{2}^{T}\left[\begin{array}{c} x^{MF}\\ x^{MLP} \end{array}\right]\right)$$

where $H_{2}$ and σ represent the weight and activation function of full connection layer II, respectively.

### 3.4. Ranking and Learning

Next, we map score $x_{ui}$ to probability ${\hat{y}}_{ui}$ by the softmax function. The probability ${\hat{y}}_{ui}$ that item i ranks first in the list of user u is defined as follows:

(16) $${\hat{y}}_{ui}=softmax\left({\hat{x}}_{ui}\right)$$

The A-DNR model focuses on top-N recommendations, so the probability of items appearing in the user list is defined as:

(17) $$P(l_{u}|\theta )=\underset{i\in l_{u}^{+}}{\prod }{\hat{y}}_{ui}\underset{k\in l_{u}^{-}}{\prod }\left(1-{\hat{y}}_{uk}\right)$$

where Θ represents model parameter vector; $l_{u}^{+}$ and $l_{u}^{-}$ represent the user u rated and unrated item sets in the recommendation list $l_{u}$, respectively.

Overfitting is a permanent problem in machine learning model optimization [33]. Attention mechanism network can enhance the feature extraction ability of the model, but too many input features will make the model easier to overfitting during training. For A-DNR model, we adopt two schemes to prevent overfit, dropout and regularization, which have been widely used in neural network models [28]. Dropout is to randomly delete some neurons during training, which has been proved to reduce the complex co-adaptation relationship of neurons in training data [34]. We introduce dropout into the feature interaction layer of A-DNR model and set its parameter to 0.5. It is worth noting that dropout is only added to the MLP of the feature interaction layer because we found in the experiment that the combined use of dropout in the feature interaction layer and attention layer will lead to some stability problems and reduce the performance of the model.

In addition, we added regularization when using cross entropy to evaluate the loss. The loss function is as follows:

(18) $$l\left(y,\hat{y}\right)=-\overset{N}{\underset{u=1}{\sum }}\left(\underset{i\in l_{u}^{+}}{\sum }\log y_{ui}+\underset{k\in l_{u}^{-}}{\sum }\log\left(1-{\hat{y}}_{ui}\right)\right)+\lambda ||\theta ||$$

where y and $\hat{y}$ represent the real value and predicted value, respectively; l(⋅) represents the binary cross entropy loss; λ represents the regularization coefficient set in the experiment; $\theta =\left\{A_{u},p_{i},h,W,H_{1},H_{2},c,b\right\}$, denotes the set of parameters in the model.

## 4. Experiment

In this section, we first introduce the experimental settings. Then, we conduct detailed experiments to answer the following questions:

- How does A-DNR perform compared with other methods?
- What is the impact of using attention mechanism to model user preferences on the model?
- How do users’ long-term preferences and short-term preferences affect the model respectively?
- How do model parameters affect the top-k recommendation performance?

### 4.1. Experimental Settings

#### 4.1.1. Datasets

We evaluated our method on three public data sets: MovieLens100K, Movielens1M, and Yahoo Movies. For these datasets, each user has at least 20 interactions with items and each item has at least 1 interaction with users. All datasets have high sparsity, which can more objectively evaluate the performance of each model. The statistics of these three datasets are summarized in Table 1.

#### 4.1.2. Comparison Methods

The following briefly introduces some comparison methods to evaluate the performance of our model:

- BPR: BPR [7] focuses on implicit feedback and optimizes personalized ranking with a pairwise loss function.
- AFM: AFM [28] is an improvement based on FM, which learns the importance of each feature interaction from data through the attention network.
- DeepCF: DeepCF [19] is a point-oriented ranking learning method, which combines representation and matching function learning-based CF methods to achieve high ranking performance.
- NeuMF: NeuMF [20] is a CF method that combines Multilayer Perceptron (MLP) and Generalized Matrix Factorization (GMF) to learn interactions between users and items.
- NAIS: NAIS [29] is a neural network CF model based on item similarity. It uses an attention mechanism network to statically model historical items, which can distinguish the importance of different historical items in the user profile and improve the quality of recommendation.
- DeepRank: DeepRank [13] focuses on top-n ranking in a list, which sets the embedding of users and items to different sizes and inputs them into MLP for ranking.
- TiSASRec: Time Interval Aware Self-Attention for Sequential Recommendation (TiSASRec) [35] proposed a time-aware self-attention mechanism, which considers not only the absolute position of the item but also the relative time interval between any two items. It is one of the state-of-the-art time interval-aware recommendation models.

#### 4.1.3. Parameters and Experimental Settings

In all methods, the learning rate and regularization coefficient (λ) are set to 0.001 and 10^−6^. The number of potential features (λ) in BPR and AFM and the embedding size of deepcf and deeprank are 16. In DeepCF, NeuMF, DeepRank and our model, the hidden layer size, training times and batch size are set to 2, 50 and 512 respectively. In AFM, TiSASRec and our method, the number of layers of attention mechanism network is set to 2.

Referring to the experimental methods of NeuMF and DeepCF, we adopt the leave-one-out to evaluate all comparison methods. To improve efficiency, we randomly selected 100 unrated products for each user as test data and reduced the list of evaluation indicators to 10. The prediction results are evaluated by two widely used evaluation indicators, Hit Ratio (HR) and Normalized Discounted Cumulative Gain (NDCG). Intuitively, the HR evaluates the accuracy of the predicted top-10 list, and NDCG highlights the top items by giving higher scores to them.

Finally, we repeat each experiment 10 times. To avoid the data error caused by model instability, we abandon the extreme value and choose the average value.

### 4.2. Overall Performance (RQ1)

We conducted detailed experiments on three data sets and compared the results with comparison methods. The experimental results are shown in Table 2, and the best performance is marked in bold.

From Table 2, we can see the following observations: (1) overall, the results of HR and NDCG are consistent. For all datasets, A-DNR, which simultaneously performs feature fusion and dynamic user preference modeling, shows superior performance than other comparison methods. (2) the performance of the proposed model A-DNR is superior to NeuMF in all three datasets, which indicates the effectiveness of using an attention mechanism network to model dynamic user preferences. In addition, A-DNR improves more obviously in the larger dataset MovieLens1M, indicating that the attention mechanism network improves model performance more obviously in the larger dataset. (3) BPR and AFM achieve limited performance compared with other comparison methods, because they are both based on traditional methods for feature interaction, lack of complex and in-depth interaction, and can not accurately grasp the deeper nonlinear features. (4) the performance of NAIS is better than other CF methods but not as good as TiSASRec. The reason is that NAIS distinguishes the importance of different historical user interactions through the attention mechanism, which improves the performance compared with other CF methods. On the other hand, TiSASRec achieves better performance than NAIS because NAIS does not consider interaction time and only models the importance of items statically, while TiSASRec models dynamic user preferences through timestamps. (5) Deeprank and DeepCF show limited performance compared with A-DNR, because they only use MLP for entity interaction fitting and do not make full use of the user-item linear features. (6) compared with A-DNR, TiSASRec has limited performance. The reason is that TiSASRec adopts the neural network to fit user-item interactions and train hyperparameters, which optimizes data input quality and improves training efficiency, but causes some loss of linear features and affect part of model performance.

### 4.3. Impact of the Attention Mechanism Network (RQ2)

Firstly, we conducted experiments to explore the influence of attention network layers on the performance of A-DNR. With other parameters unchanged, the layers of the attention network were set as 0, 1, 2, 3 and 4, respectively. Table 3 shows the experimental results. It can be observed that the model performance is significantly improved by adding attention mechanism, and the model achieves the best performance when the number of attention layers is 2. This proves the effectiveness of using attention mechanism networks to model user preferences. However, when there are too many layers of attention network, the model will have problems such as overfitting, which will limit the model performance. Therefore, we choose a two-layer attention network in the following experiments.

Next, we explored the effect of the attention mechanism on model complexity. We removed the attention mechanism part of A-DNR, and input user embedding and item embedding directly into MF and MLP for feature fusion, named DNR. Therefore, we analyzed the influence of the attention mechanism on the model by comparing A-DNR and DNR. The addition of the attention module will inevitably improve the spatial complexity of the model, so we mainly focus on the time complexity. Since different resource configurations and different operating environments will affect the training time, we kept the parameters unchanged according to the initial settings, trained DNR and A-DNR 10 times on the same machine, and counted the average training time of the two models. Table 4 shows the experimental results.

The running time of A-DNR is higher than that of DNR, but the increased time cost is not particularly significant, which proves that the addition of the attention mechanism network will slightly increase the time complexity of the model. In conclusion, considering the performance improvement, the addition of the attention mechanism has a positive impact on the model.

### 4.4. Impact of Long-Term and Short-Term User Preferences (RQ3)

Dynamic user preference modeling for the feature fusion model is a key technique proposed in our method, and a large number of experiments are carried out in this section to prove its effectiveness. Firstly, it can be seen from Table 2 that both AFM and ATRank models using the attention network produce good ranking performance. However, they directly model user preferences from all historical interactions, ignoring the specific interaction time of historical interaction items and lacking grasp of user short-term interest preference information. In contrast, our proposed model A-DNR adopts a ternary attention mechanism, which considers predicted items, recent interaction items and their interaction time to model user short-term preferences. This enables A-DNR to achieve better ranking performance than AFM and NAIS, and shows the effectiveness of user short-term preference modeling to a certain extent.

To further explore the impact of user long-term and short-term preferences on the model, we removed the user short-term preference part and long-term preference part of the A-DNR model respectively for ablation experiments, and other parts remained unchanged. The results of the ablation experiments are shown in Table 5 and Table 6, where A-DNR-L indicates that the model only models user long-term preferences, and A-DNR-S indicates that the model only models user short-term preferences. Intuitively, the modeling of user long-term preference and short-term preference at the same time can effectively improve the performance of the model. The lack of any user preference will lead to the limitation of model performance in varying degrees. The experimental results demonstrate the effectiveness and necessity of introducing the attention mechanism network to model dynamic user preferences.

Finally, we conduct experiments to analyze the comparison of user long-term and short-term preference fusion methods. The experimental results are shown in Table 7 and Table 8. Among them, A-DNR indicates that the model uses a fully connected layer to fuse the user long-term and short-term preferences, while A-DNR* indicates that the model uses a linear method to fuse the two. Intuitively, the A-DNR model achieves better model performance than the A-DNR* model. This shows that simply adopting a linear method to fuse long-term and short-term preferences cannot accurately fit each user’s interests, and lead to the loss of the overall performance of the model. At the same time, it also shows the effectiveness of modeling dynamic user preferences by fusing user long-term and short-term preferences through a fully connected layer.

### 4.5. Impact of Model Parameters (RQ3)

We conduct extensive experiments to investigate the effect of list length (k) on A-DNR. The results are shown in Table 9 and Table 10.

From Table 9 and Table 10, we first observe that adding more item information to the model can further improve the performance of the model. As the list length K increases, the values of HR and NDCG also increase. However, an increase in the length of the list will inevitably lead to a large increase in the complexity of the model, which greatly increases the training time of the model. Therefore, we propose to set K to 5, which can save training time while maintaining performance.

To fully exploit the potential of A-DNR, we explore the impact of the depth of hidden layers in the feature interaction layer on model performance. In this experiment, the size of the hidden layer is set to [8], [16,8], [32,16,8], [64,32,16,8], [128,64,32,16,8], and other parameters remain unchanged. The experimental results are shown in Table 11.

As shown in Table 11, when L = 2, A-DNR achieves the best performance on both datasets. At L < 3, as the number of hidden layers increases, the values of HR@10 and NDCG@10 of the model also increase. When L ≥ 3, the values of HR@10 and NDCG@10 of the model decrease as the depth of the hidden layer increases. To sum up, proper hidden layer depth can improve the performance of the model.

## 5. Conclusions 

In this work, we explore the feasibility of feature fusion models based on dynamic user preferences for ranking recommendations. We design a two-stage recommendation model, A-DNR, which includes attention-based dynamic user preference modeling and feature fusion-based list ranking recommendation. Experiments show that introducing the attention mechanism to model dynamic user preferences can significantly improve the performance of the feature fusion model. In addition, we also explore the impact of the attention mechanism network on the model through extensive experiments and demonstrate the effectiveness of dynamic user preference modeling for feature fusion models.

A-DNR models dynamic user preferences through the fusion of user long-term and short-term preferences. To facilitate training, this paper treats user embeddings as user long-term preferences and does not model long-term interaction items separately. In future work, we will explore an efficient method to model long-term user preferences and consider adding more auxiliary information, such as knowledge graph and comment information, to enhance feature representation and improve model performance.

## Figures and Tables

**Table 1:** Statistics of the datasets.

Statistics	MovieLens100K	MovieLens1M	Yahoo Movies
# of users	943	6040	7642
# of items	1682	3952	11,915
# of ratings	100,000	1,000,000	211,231
# Sparsity	0.9369	0.9553	0.9977

**Table 2:** Performance comparison of different comparison methods.

Methods	MovieLens100K		MovieLens1M		Yahoo Movies	
	HR@10	NDCG@10	HR@10	NDCG@10	HR@10	NDCG@10
BPR	0.6958	0.4257	0.6852	0.4227	0.7462	0.6325
AFM	0.7059	0.4339	0.7026	0.4318	0.7528	0.6431
DeepCF	0.7126	0.4437	0.7251	0.4416	0.7913	0.6851
NeuMF	0.7169	0.4526	0.7292	0.4478	0.7923	0.6825
NAIS	0.7202	0.4565	0.7333	0.4596	0.8001	0.6922
DeepRank	0.7670	0.4739	0.7555	0.5011	0.8111	0.6978
TiSASRec	0.7989	0.5217	0.8004	0.5391	0.8132	0.7067
A-DNR	**0.8196**	**0.5326**	**0.8246**	**0.5512**	**0.8322**	**0.7159**
Improvement	2.5%	2.1%	3.0%	2.2%	2.3%	1.3%

**Table 3:** The impact of the depth of attention layer.

t	MovieLens100K		MovieLens1M	
	HR@10	NDCG@10	HR@10	NDCG@10
0	0.8039	0.5224	0.7987	0.5443
1	0.8068	0.5290	0.8194	0.5492
2	**0.8135**	**0.5321**	**0.8229**	**0.5682**
3	0.8003	0.5145	0.8069	0.5217
4	0.7192	0.4784	0.7268	0.5046

**Table 4:** Time cost on DNR and A-DNR.

	MovieLens100K	MovieLens1M
DNR	4 m 49 s	35 m 35 s
A-DNR	5 m 31 s	42 m 15 s

**Table 5:** The impact of user long-term and short-term preferences on HR@10.

	MovieLens100K	MovieLens1M
A-DNR-L	0.7912	0.7321
A-DNR-S	0.7855	0.7498
A-DNR	**0.8131**	**0.8231**

**Table 6:** The impact of user long-term and short-term preferences on NDCG@10.

	MovieLens100K	MovieLens1M
A-DNR-L	0.5234	0.4535
A-DNR-S	0.4987	0.4345
A-DNR	**0.5319**	**0.5683**

**Table 7:** The impact of user long-term and short-term preference fusion method on HR@10.

	MovieLens100K	MovieLens1M
A-DNR*	0.7821	0.8013
A-DNR	**0.8131**	**0.8231**

**Table 8:** The impact of user long-term and short-term preference fusion method on NDCG@10.

	MovieLens100K	MovieLens1M
A-DNR*	0.5216	0.5465
A-DNR	**0.5319**	**0.5683**

**Table 9:** The impact of list length on HR@10.

K	MovieLens100K	MovieLens1M
2	0.7910	0.7952
5	0.8103	0.8216
10	0.8133	0.8273
15	0.8209	0.8294

**Table 10:** The impact of list length on NDCG@10.

K	MovieLens100K	MovieLens1M
2	0.5169	0.5358
5	0.5326	0.5662
10	0.5425	0.5685
15	0.5495	0.5699

**Table 11:** The impact of hidden layer depth of MLP.

L	MovieLens100K		MovieLens1M	
	HR@10	NDCG@10	HR@10	NDCG@10
1	0.7972	0.5250	0.7994	0.5322
2	**0.8136**	**0.5326**	**0.8276**	**0.5662**
3	0.8043	0.5267	0.8134	0.5517
4	0.7462	0.4817	0.8067	0.5406
5	0.7414	0.4682	0.7889	0.5339

## Data Availability

Not applicable.
